# Time-lapse angiography of the ocular fundus: a new video-angiography

**DOI:** 10.1186/s12880-019-0398-1

**Published:** 2019-12-19

**Authors:** Chufeng Gu, Jili Chen, Tong Su, Qinghua Qiu

**Affiliations:** 1 Department of Ophthalmology, Shanghai General Hospital, Shanghai Jiao Tong University School of Medicine; Shanghai Key Laboratory of Ocular Fundus Diseases; Shanghai Engineering Center for Visual Science and Photomedicine, Shanghai, 200080 People’s Republic of China; 2 Department of Ophthalmology, Shibei Hospital of Jing’an District, Shanghai, 200040 People’s Republic of China; 3 Department of Ophthalmology, Shigatse People’s Hospital, Xizang, 857000 People’s Republic of China

**Keywords:** Time-lapse photography, Ocular fundus angiography, Three-dimensional visualization, Dynamic report

## Abstract

**Background:**

Ocular fundus angiography is an indispensable component of the tests utilized for fundus diseases. Dynamic angiography results can provide additional information; however, many difficulties remain. In this study, we introduce a modified method, time-lapse angiography (TLA), to dynamically present imaging results.

**Methods:**

TLA, combining time-lapse photography and fundus angiography (using Heidelberg retina angiography II, Germany), includes pre-photographing and post- photosynthesis and ultimately produces a video that is approximately 15 s in length.

**Results:**

Four typical videos in the article showed the characteristics of TLA, including a short and rapid but continuous and integral presentation, highly valid information, high definition, etc.

**Conclusions:**

TLA is beneficial for the diagnosis of diseases and the assessment of progression and is convenient for peer communication, patient interpretation, and student education. The application of time-lapse photography in ocular fundus angiography is a monumental and innovative attempt.

## Background

Fluorescein fundus angiography (FFA) and indocyanine green angiography (ICGA) are indispensable components of the tests utilized for fundus diseases. Conventionally, imaging results are recorded by an ophthalmic medical technician, who selects a few typical images and uses a paper-based imaging report to present the results. This downgrades the dynamic changes in eye-ground vessels into isolated and static ones. Additionally, the amount of information presented to doctors is greatly reduced. Thus, determining how to record the complete angiography process is highly important.

One of the best devices to use is a Heidelberg retina angiography II (HRA II, Heidelberg Engineering, Heidelberg, Germany), which can record short, one-minute videos, but the imaging process takes more than 10 min. In addition, being able to video-record the entire process, store data and deliver traditional videos is not easy when dealing with high-volume data, which contains too much invalid information. Consequently, if we could invent new technology to solve these problems, we could improve the process.

Time-lapse photography is a recording technique that captures images at a much lower frequency than the frequency that is applied to view the sequence. As a result, when played at a normal speed, time seems to move faster, which creates the lapse. Currently, there are a variety of applications for this technology in the medical field, such as for the diagnosis of human preimplantation embryo viability [1] and the quantification of bone resorption and formation [2]. In ophthalmology, time-lapse photography has been used to illustrate the development of idiopathic macular holes and the effects of surgical treatment [3]. However, applying time-lapse photography in ocular fundus angiography to assist in the diagnosis of ophthalmic diseases has not been reported.

We wish to introduce a modified method, time-lapse angiography (TLA), that combines time-lapse photography and fundus angiography (using HRA II) to dynamically present imaging results.

## Methods

TLA includes pre-photographing and post-photosynthesis. First, we took 3–5 fundus photographs before injecting fluorescein. Then, we used time-lapse photography to take at least 20 images per minute from the beginning of the angiography process, containing the pre-arterial phase, retinal arterial stage, arterial-venous stage, venous stage, and late-venous stage. In principle, we needed more than 300 images. After removing the blurry images caused by moving and blinking of the eyes, we used the following software to process and synthesize the remaining photos: LRTimelapse (Germany), Adobe Photoshop Lightroom (Adobe Systems Incorporated, USA), and Adobe After Effects (Adobe Systems Incorporated, USA).

## Results

**Fig. 1:**
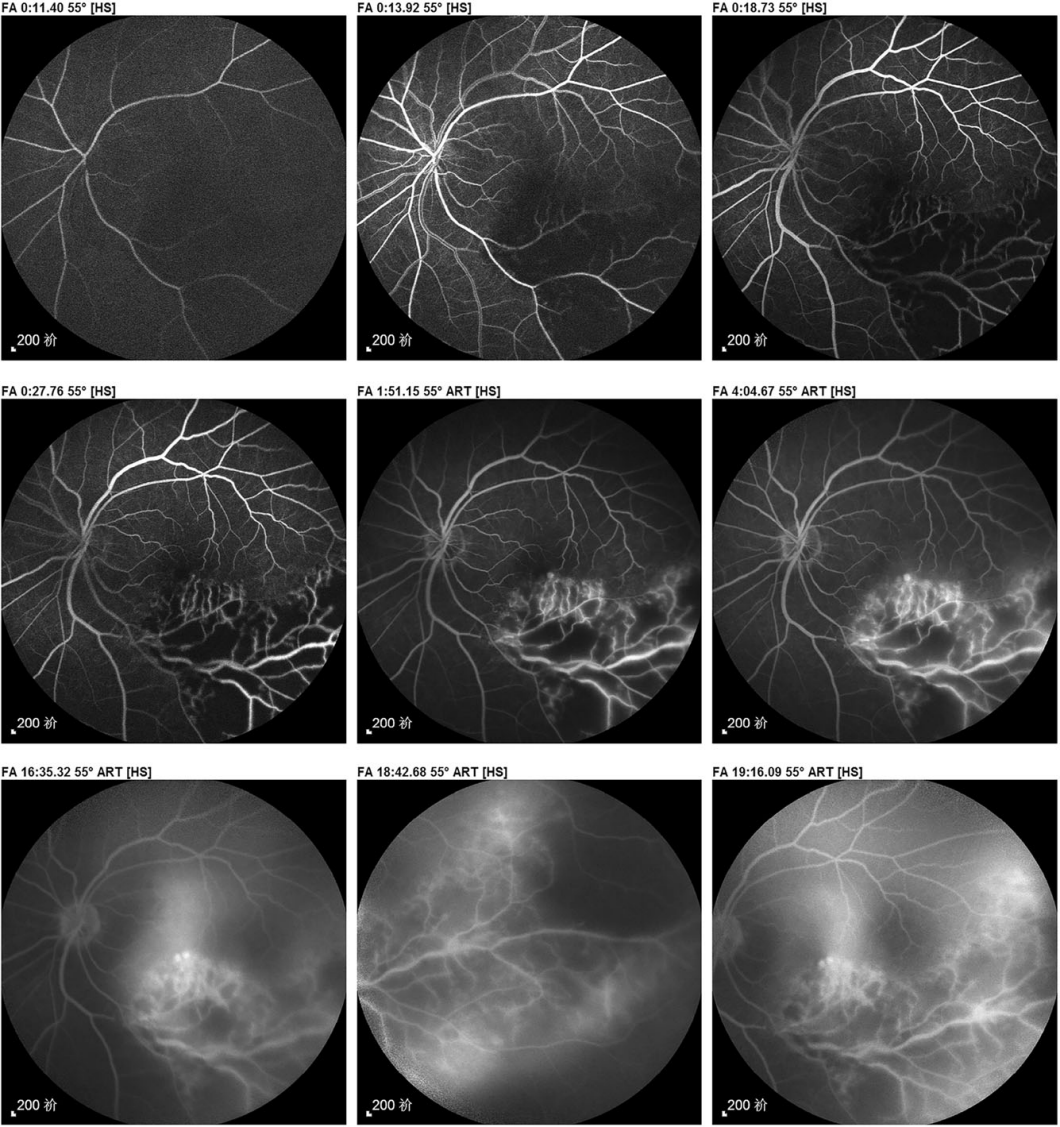
The paper-based imaging report corresponding to Video S1

In the 12 s stereoscopic video (Video S1, Additional file 1), we observed that fluorescein leaked from the retinal vessels to the outside and eventually into the vitreous cavity in the late staining stage. This suggested that the lesion had already affected the vitreous cavity. However, this phenomenon may be confused with retinal vascular leakage in a planar and static report (Fig. 1).

**Fig. 2:**
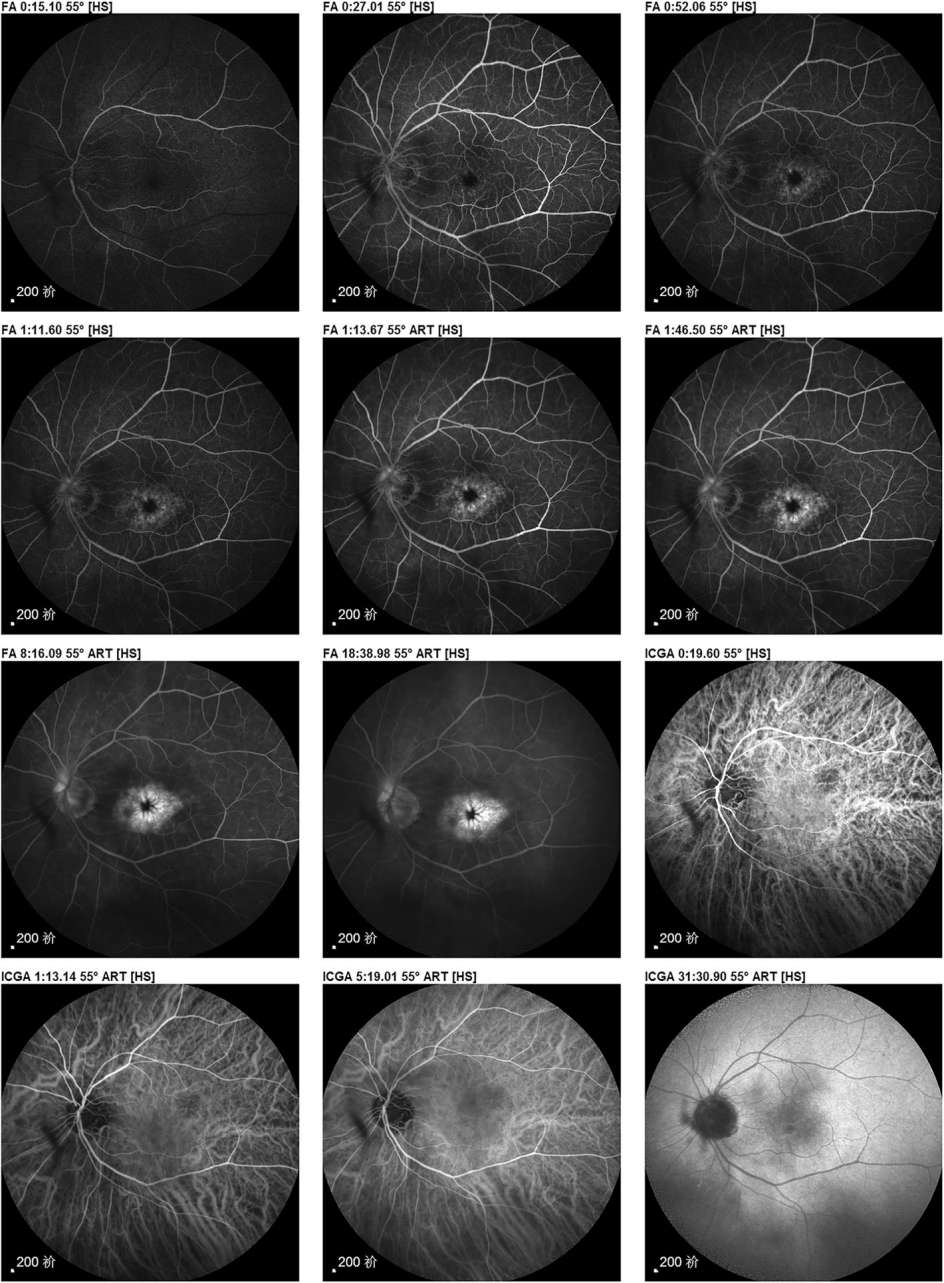
The paper-based imaging report corresponding to Video S2

Video S2 (Additional file 2), approximately 17 s in length, specifically and representatively shows the entire fluorescence filling process of cystoid macular edema: the blurry leakage sites of the microvessels around the fovea became increasingly larger in the late-venous stage, fusing with each other and forming petal-like or radial appearance spots around the fovea. This is especially suited for teaching. Furthermore, we discovered that a flocculent shadow existed in front of the retina throughout these stages, which represents the performance of the vitreous opacity; however, this finding is likely to be considered hypofluorescence in a planar, paper-based report (Fig. 2).

**Fig. 3:**
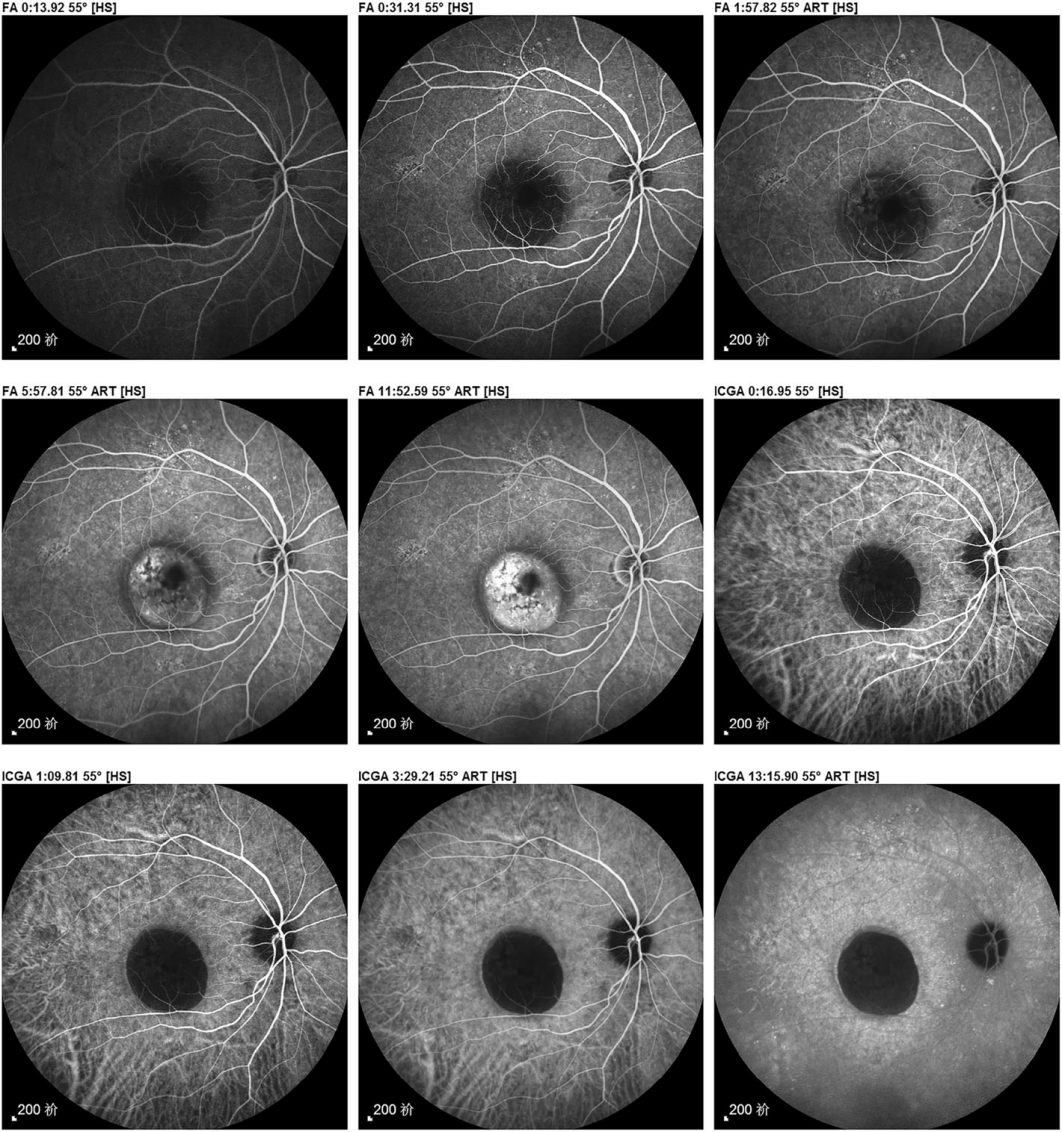
The paper-based imaging report corresponding to Videos S3 and S4

Video S3 (20 s, Additional file 3) and Video S4 (19 s, Additional file 4) are the results of FFA and ICGA, respectively, for retinal pigment epithelium detachment — similar to the evolution of stars and the eternity of black-holes — which fully embodies the artistic beauty of video-angiography. The corresponding paper-based imaging report was shown in Fig. 3.

## Discussion

Just as films have developed from 2D to 3D to Imax, and as the form of artistic expression has changed from a flat surface into a three-dimensional one, the development of ocular fundus angiography reports from static and planar to dynamic and stereoscopic is an inevitable trend. This study introduces TLA to stereoscopically present imaging results. Although TLA only combines two established methods, this innovation is beneficial for the diagnosis of diseases and the assessment of progression. TLA reconnects static images and turns fundus angiography into a real dynamic check, which brings the three-dimensional aspect of imaging results to life (Videos S1 and S2). Moreover, TLA solves the deficiency of traditional videos with low resolution and high-volume data (within 20 s). This new video-angiography maintains the advantages of high definition in the paper-based reports and massive amounts of valid information in the conventional video. We can choose the appropriate window of time to observe dynamic changes in specific parts of fundus vessels, especially for the vasa vasorum and new vessels. In addition to facilitating a doctor’s diagnosis, this dynamic report is convenient for peer communication, patient interpretation, and student education. Furthermore, this technique is applicable to other areas of medical angiography and may bring new inspiration to the development of imaging technology. However, further studies with a large sample and quantitatively statistical analysis are necessary to get more objective results.

## Conclusions

TLA is beneficial for the diagnosis of diseases and the assessment of progression, and it is convenient for peer communication, patient interpretation, and student education. The application of time-lapse photography in ocular fundus angiography is a monumental and innovative attempt.

## Supplementary information

**Additional file 1. Video S1:** TLA of fundus diseases involving the vitreous cavity.

**Additional file 2.** **Video S2:** TLA of cystoid macular edema.

**Additional file 3. Video S3:** TLA of FFA for retinal pigment epithelium detachment.

**Additional file 4. Video S4:** TLA of ICGA for retinal pigment epithelium detachment.

## Data Availability

The datasets used and analysed during the current study are available from the corresponding author on reasonable request.

## Abbreviations

FFA: Fluorescein fundus angiography
HRA II: Heidelberg retina angiography II
ICGA: Indocyanine green angiography
TLA: Time-lapse angiography
